# Performance Improvement of Residue-Free Graphene Field-Effect Transistor Using Au-Assisted Transfer Method

**DOI:** 10.3390/s21217262

**Published:** 2021-10-31

**Authors:** Yamujin Jang, Young-Min Seo, Hyeon-Sik Jang, Keun Heo, Dongmok Whang

**Affiliations:** 1 School of Advanced Materials Science and Engineering, Sungkyunkwan University, Suwon 16419, Korea; cyon24@skku.edu; 2 Institute of Advanced Composite Materials, Korea Institute of Science and Technology (KIST), Jeonju 55324, Korea; ymseo88@kist.re.kr (Y.-M.S.); hsjang@kist.re.kr (H.-S.J.); 3 School of Semiconductor and Chemical Engineering, Jeonbuk National University, Jeonju 54896, Korea; kheo@jbnu.ac.kr

**Keywords:** graphene, field-effect transistor, graphene transfer, electrical property

## Abstract

We report a novel graphene transfer technique for fabricating graphene field-effect transistors (FETs) that avoids detrimental organic contamination on a graphene surface. Instead of using an organic supporting film like poly(methyl methacrylate) (PMMA) for graphene transfer, Au film is directly deposited on the as-grown graphene substrate. Graphene FETs fabricated using the established organic film transfer method are easily contaminated by organic residues, while Au film protects graphene channels from these contaminants. In addition, this method can also simplify the device fabrication process, as the Au film acts as an electrode. We successfully fabricated graphene FETs with a clean surface and improved electrical properties using this Au-assisted transfer method.

## 1. Introduction

Since graphene was mechanically exfoliated from graphite in 2004, the material’s attractive mechanical, optical, and electrical properties have stimulated a great deal of related research [1,2,3,4]. The mechanical exfoliation using highly oriented pyrolytic graphite (HOPG) was widely used in the early stages of graphene research. The exfoliated graphene demonstrates nearly ideal characteristics, and its maximum electron mobility has been reported to be as high as 230,000 cm^2^/V·s [5]. Utilizing the electrical properties of graphene with high mobility, it can be used for applications such as high-speed optical sensors [6,7], gas and chemical sensors [8,9], and transparent conducting electrodes [10,11]. Unfortunately, however, graphene cannot be obtained in large-area with the method, which posed a serious barrier to realizing practical applications of graphene. To resolve this problem, a number of methods capable of producing large-area graphene were developed, including high-temperature epitaxy growth [12,13], chemical reduction [14], and chemical vapor deposition (CVD) [15,16]. While synthesis via CVD is capable of producing large-area monolayer graphene at a lower price and of higher quality than the alternatives [15], the electrical properties of CVD graphene include relatively low mobility, a high Dirac point, and high carrier density, all of which make it inferior to exfoliated graphene [17,18]. These shortcomings of CVD graphene are due to a partially generated overlapped multi-layer and a higher density of defects, both of which result from the synthesis process and catalysts [18,19].

More recently, the incorporation of new catalysts and optimizations into the CVD process has allowed for the synthesis of single-crystal graphene that does not suffer from these problems [20,21]. However, extrinsic factors remain degrading the electrical performance of graphene, the most critical of which is the organic contamination generated during the transfer process. Most commonly, an organic supporting film such as poly(methyl methacrylate) (PMMA) is used to transfer graphene. Unfortunately, it is almost impossible to completely remove the organic film residue, which results in the absorption of H_2_O and O_2_ and consequently p-doping of graphene [22,23,24].

Various processes are employed to eliminate this extrinsic factor. The most widely used method is vacuum thermal annealing at 300~400 °C in ambient H_2_/Ar [25,26,27,28]. A number of researchers have tested this method and confirmed a reduction of PMMA residues and an associated improvement in the resulting grapheme’s electrical properties [22,28,29,30]. Other researchers have employed alternative polymer films in the transfer process, such as polycarbonate [23,29] or photoresist (PR) [31]. However, as these methods still do not completely remove all of the organic residues, a more reliable solution is needed. Recently, a novel method of separating catalyzed metal and graphene by inducing an electric charge has been reported [32]. While this method makes it possible to transfer a relatively large area of graphene without the risk of contaminant residues, it is limited in how it can be applied to a conducting substrate or a roll-to-roll process.

In this study, we propose a novel transfer method that resolves the drawbacks associated with conventional approaches. We completely avoid organic contamination by transferring graphene after a thin Au film was deposited on the graphene as-grown on a Cu substrate. As a noble metal, Au prevents any property changes to the graphene and can be easily etched using an ionic solvent. We transferred graphene onto the target substrate via this method and then analyzed its surface using Raman spectroscopy and X-ray photoelectron spectroscopy (XPS). We confirmed the removal of organic residues by evaluating the electrical properties of a graphene field-effect transistor (GFET) fabricated using the proposed method. Ultimately, we report the fabrication of a GFET with enhanced electrical properties and a clean graphene surface.

## 2. Materials and Methods

### 2.1. Graphene Synthesis and Transfer Process

Graphene was grown on Cu foils by CVD. After annealing a Cu foil at 1000 °C for 15 min, graphene was synthesized for 30 min with CH_4_ (25 sccm) and H_2_ (10 sccm) gas at 1000 °C. After growth, the as-grown graphene layer on Cu foil was cut into two parts and used for following PMMA- and Au-assisted transfer. Au film (30 nm) was deposited on the as-grown graphene substrate by thermal evaporator or PMMA (0.01%, Sigma-Aldrich, Burlington, MA, USA) was coated by spin coater for comparison. The backside graphene of Cu foil was removed by oxygen plasma treatment for 10 s at a power of 10 W. The Cu foil underlying the graphene layer was removed by floating on an aqueous solution of (NH_4_)_2_S_2_O_8_ for 1 h. Graphene supported on Au or PMMA layer was cleaned three times by floating on DI water for 30 min. 

### 2.2. GFET Fabrication Process

**Figure 1:**
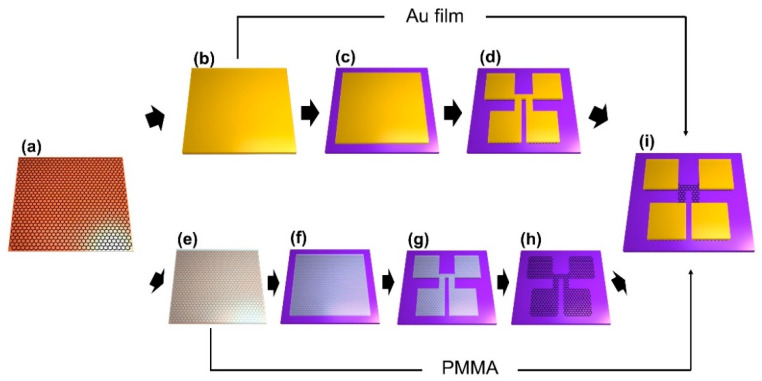
Schematic of graphene FET fabrication. (**a**) Growth of graphene on Cu film, (**b**) Thermal evaporation of Au (30 nm) on the graphene/Cu, (**c**) Transfer of Au/graphene on SiO_2_/Si substrate after chemical etching of underlying Cu film, (**d**) Photolithographic patterning of the Au/graphene with 4-terminal Hall-bar structure, (**e**) PMMA coating on the graphene/Cu, (**f**) Transfer of PMMA/graphene after Cu etching and subsequent PMMA removal (**g**) Photolithographic patterning of the graphene with 4-terminal Hall-bar structure, (**h**) Etching of the unpatterned graphene by O_2_ RIE, and (**i**) Fabrication of GFET with exposed graphene channel. For graphene transferred by Au assisted method, the Au layer on the channel was selectively etched. For graphene transferred by PMMA assisted method, Cr/Au (5/30 nm) were deposited on the electrode area.

Figure 1 schematically shows two GFET fabrication processes using the proposed Au-assisted transfer and conventional PMMA-assisted transfer methods [33]. The conventional wet transfer method includes the coating of graphene with organic PMMA film, transfer of the PMMA coated graphene layer onto an arbitrary substrate after Cu foil is removed with a Cu etchant, and removal of the PMMA film on graphene (Figure 1a,e,f). In this approach, primary surface contamination will occur in the form of residues from the organic supporting film. In contrast, the proposed method uses Au as a supporting film instead of an organic film (Figure 1b,c). The Au film blocks all organic residues induced in the graphene transfer process and can be easily removed later with an Au etchant solution. In addition, the use of Au dramatically reduces processing time and eliminates contamination during the GFET fabrication. 

The process of GFET fabrication using PMMA assisted transfer includes device patterning on the graphene by photo-lithography processes and O_2_ plasma etching and finally depositing a metal electrode (Figure 1g–i). Here again, photoresist (PR) and solvent may contaminate the graphene, and the organic residues will increase the contact resistance between the electrode and graphene [34]. However, our Au-assisted graphene transfer method enables a comparatively simple fabrication process, and more importantly, prevents organic contamination at the Au-graphene interface. The fabrication process using Au-assisted transfer involves patterning the device structure on the transferred Au/graphene, removal of unpatterned Au film by a dip in Au etchant (1:4:40 I_2_/KI/H_2_O), and O_2_ reactive ion etching (RIE) to etch unwanted graphene. Finally, residue-free GFETs are fabricated through the chemical etching of Au film at the channel area Au film (Figure 1d,i).

### 2.3. Measurement Equipment and Condition

Scanning electron microscopy (SEM) images were acquired with a JEOL JSM-6701F field emission scanning electron microscope (FESEM). Raman spectroscopy (Renishaw, RM-1000 Invia) with the wavelength of 532 nm (Ar-ion laser) was used to characterize the graphene on 300 nm SiO_2_ substrates. XPS spectra were collected using a monochromatic Al Kα *X*-ray source and Omicron EA125 hemispherical analyzer. The graph fitting method is Lorentz-Gaussian fitting. Atomic force microscopy (AFM) images were acquired with a Park System NX10 using non-contact mode. 

## 3. Results and Discussion

**Figure 2:**
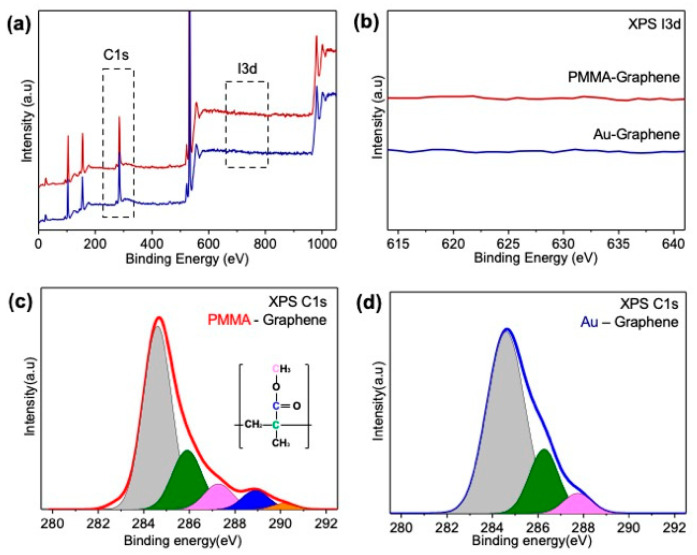
(**a**) XPS data for graphene transferred using PMMA and graphene transferred using Au film. (**b**) The I_3d_ region in (**a**). (**c**) C_1s_ region in graphene transferred using PMMA. (**d**) C_1s_ region in graphene transferred graphene using Au film.

Figure 2 presents the XPS results for the graphene layers transferred onto SiO_2_/Si substrates using the Au-assisted and the PMMA-assisted methods. XPS in a wide area (beam diameter ~8 mm) can detect not only the C-C sp^2^ hybridized carbon of graphene layer but also sp^3^ carbon peaks caused by organic contamination on the graphene′s surface. Through this XPS analysis, the effects of the supporting film and the etchant used to remove the supporting film on the graphene surface were compared (Figure 2a). Since iodine, the main component of Au etchant, can act as a p-type surface dopant of graphene layer [35], and the effects of the Au etchant on graphene were analyzed (Figure 2b). XPS spectra of the graphene layers, whether transferred using PMMA or Au film, showed no peaks related to iodine or Cu, suggesting that Cu film and iodine molecules were completely removed and did not affect the graphene layer (Figure S1 in Supplementary Material). Figure 2c,d are the enlarged and fitted graphs of the C_1_ region in Figure 2a; the carbon bonds of graphene or other functional groups are shown in the inset [28]. These two graphs illustrate that Au-transferred graphene exhibits a larger sp^2^ carbon peak than PMMA-transferred graphene and that contamination from organic residue was lower in Au-transferred graphene. In particular, the peak related to the carboxyl functional group was only observed in PMMA-transferred graphene, not Au-transferred graphene. The carboxyl group PMMA-transferred graphene was probably generated either in the acetone used to remove the PMMA or in the PMMA itself. We also note that the carboxyl group degrades graphene’s electrical properties and leads to p-doping and a decrease in carrier mobility of the graphene layer [36,37].

To highlight the performance improvements by our proposed approach, we fabricated back-gated GFETs using PMMA-assisted and Au-assisted transfer methods. Previously, improvement of the contact resistance of graphene using Au assisted transfer similar to our approach [34]. However, we directly measured the sheet resistance by Hall-bar structure with gate bias. As a result, it can show more information about the GFETs. A Hall-bar structure device was used to measure the carrier mobility of the graphene [38]. Sheet resistance (*R_sh_*) was also calculated using the following Equation (1):

(1) $$R_{sh}=\frac{(V_{x_{2}}-V_{x_{1}})w}{I\cdot d}$$

where *w* is the width of the Hall-bar-channel, *d* is the distance between the voltage leads *x*_2_ and *x*_1_, *I* is the current flowing between *x*_2_ and *x*_1_. Carrier density (*n_s_*) values can be obtained from Equation (2):

(2) $$n_{s}=\frac{C_{ox}(V_{g}-V_{dirac})}{q}$$

where *C_ox_* is gate oxide capacitance, and *q* is Coulomb unit charge. Based on these values, mobility can be obtained using Equation (3):

(3) $$\mu =-\frac{1}{n_{s}eR_{sh}}$$

**Figure 3:**
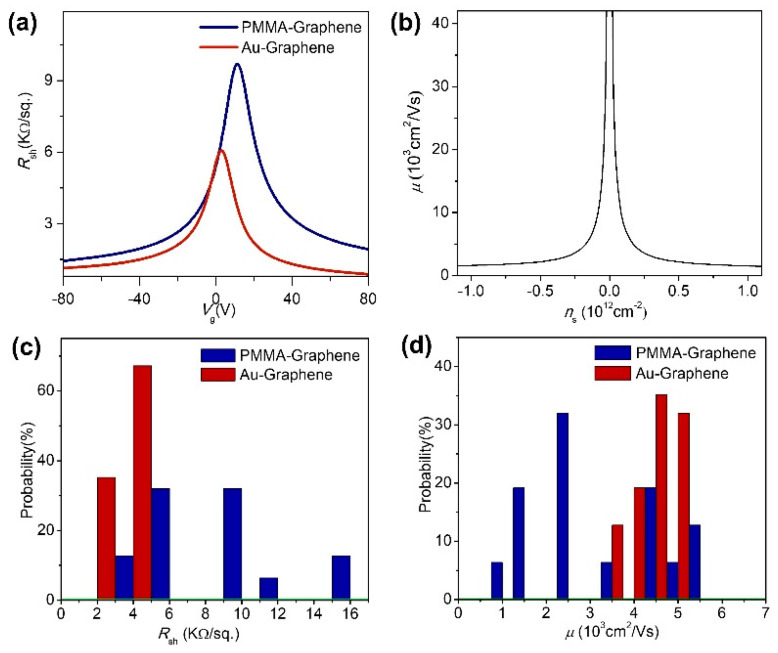
(**a**) Gate voltage (*V_g_*) vs. sheet resistance (*R_sh_*) curves at GFETs with a Hall-bar structure, (**b**) A calculated carrier density (*n_s_*) vs. carrier mobility (*μ*) curve of the GFET fabricated by Au-assisted transfer method, (**c**) The distribution of measured sheet resistance at *V*_dirac_ and (**d**) The distribution graph of mobility at *n*_s_ = 3 × 10^11^ cm^−2^.

The channel width (*w*) of our Hall-bar device was 5 µm, and the length between *x*_2_ and *x*_1_ was 20 µm. Devices were measured at room temperature with a high vacuum condition of 1 × 10^−6^ Torr. Figure 3a shows the sheet resistances of the GFETs calculated using Equation (1). GFETs fabricated using PMMA-assisted transfer had a Dirac point at a higher positive voltage than GFETs using Au-assisted transfer. Figure 3b presents carrier mobility vs. carrier density curve, which was calculated using Equation (3) based on the sheet resistance of Au-transferred graphene in Figure 3a. Using 32 GFETs simultaneously fabricated on a SiO_2_/Si substrate for each of PMMA and Au-transfer graphenes, we determined the distribution of sheet resistance at the Dirac point and the mobility distribution at *n_s_* = 3 × 10^11^ cm^−2^. While the average sheet resistance of the Au-transferred GFETs was 4300 ± 400 Ω/sq., PMMA-transferred GFETs had a higher sheet resistance of 8400 ± 3300 Ω/sq. (Figure 3c). With respect to mobility, Au-transferred GFETs had a value of 4600 ± 400 cm^2^/V·s, while PMMA-transferred GFETs had a value of 2800 ± 1300 cm^2^/V·s (Figure 3d). In addition, sheet resistance and mobility were uniform throughout the Au-transferred graphene but uneven in PMMA-transferred graphene. 

Direct comparison of the channel was possible in our GFET measurement process, as the value of contact resistance was similar to that of sheet resistance [39]. In other words, XPS observations confirmed the presence of a large amount of residue, including the carboxyl group, on the PMMA-transferred graphene surface but no residues on the Au-transferred graphene. SEM, AFM, and optical images of the device′s channels further confirm that the graphene channel fabricated using Au-based process is clean, but the channels fabricated with PMMA assisted transfer had many organic residues (Figures S2 and S3 in Supplementary Material). As is known, the organic residue of PMMA causes p-type doping, and the Dirac point is located at a positive gate voltage, but when PMMA is not used, the Dirac point shifts toward 12 V to 3 V (Figure 3a) [22,40]. These residues are dispersed around the graphene irregularly and have an electro-scattering effect, resulting in lower average value and non-uniform distribution of carrier mobility of PMMA-transferred graphene devices. The electrical characteristics of the GFETs confirm that the Au-assisted graphene transfer enables the graphene device without organic residues, pointing a way for the fabrication of a graphene device with superior electrical properties and a cleaner surface. 

**Figure 4:**
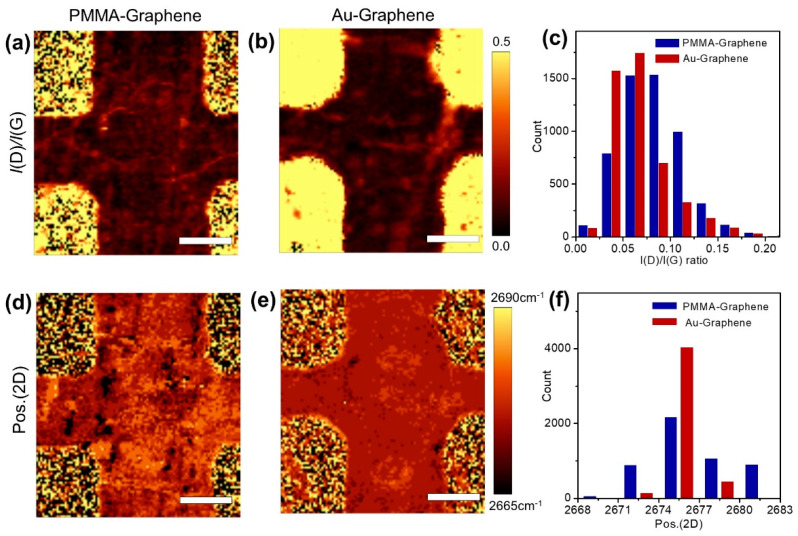
The *I*(D)/*I*(G) Raman mapped image of (**a**) graphene transferred using PMMA and (**b**) graphene transferred using Au film. (**c**) The *I*(D)/*I*(G) ratio distribution graph. The 2D Raman mapped image of (**d**) graphene transferred using PMMA and (**e**) graphene transferred using Au. (**f**) The 2D position distribution graph. Scale bars, 5 μm.

Raman mapping of the graphene channels of the GFETs provided the most detailed analysis. The distribution of organic residue on the graphene surface can be characterized by the intensity ratio of D band (~1350 cm^−1^) and G band (~1580 cm^−1^) in the Raman mapping image (Figure S4 in Supplementary Materials). In general, a higher *I*(D)/*I*(G) value indicates a higher defect density in the graphene area [41]. PMMA-transferred graphene often has high D band intensity of amorphous carbon [28], and the ratio of D and G peaks varies depending on the organic residue on the graphene [42]. Figure 4a,b presents Raman maps of the *I*(D)/*I*(G) of our GFETs. Compared with the mapping image of the GFET transferred with Au, *I*(D)/*I*(G) values are non-uniform in the GFET transferred to PMMA. In addition, a distribution graph of *I*(D)/*I*(G) on graphene shows that the average *I*(D)/*I*(G) value is also larger in the GFET transferred with PMMA (Figure 4c).

The channel map of the GFET fabricated using PMMA reflects a broad distribution of 2D position, while the channel map of the Au-using GFET is relatively uniform (Figure 4d–f). Additionally, Figure 4f shows that the average 2D peak position of graphene transferred with PMMA is blue-shifted than that of graphene transferred with Au, indicating non-uniform p-type doping of GFET fabricated with PMMA (Figure S4c,f in supplementary material). These results confirm that graphene transferred with Au film maintains a higher degree of surface reliability throughout the whole fabrication process.

## 4. Conclusions

In this paper, we compared graphene transferred using Au and PMMA via XPS and Raman mapping. We also examined the electrical properties of back-gated GFETs fabricated with both methods. As a result, it was confirmed that the graphene transferred by the Au-assisted transfer has a cleaner and more uniform surface than the graphene transferred using the conventional organic supporting film. The electrical characteristics of the graphene transferred with PMMA and Au film were measured by fabricating Hall-bar type back-gated GFETs. The GFET fabricated using Au-transferred graphene exhibited an average sheet resistance of 4000 Ω/sq and average mobility of 4500 cm^2^/V·s, which are much improved values than the characteristics of devices fabricated from PMMA-transferred graphene. This proposed Au-assisted approach for fabricating GFETs may have significant practical value in the development of graphene-based device applications.

## Data Availability

Not applicable.

## Supplementary Materials

The following are available online at https://www.mdpi.com/article/10.3390/s21217262/s1, Equation S1: Calculation of Electronic property, Figure S1: XPS spectra of transferred graphenes, Figure S2: Optical and SEM images of graphene channels, Figure S3: AFM images of graphene channels in the fabricated GFETs, Figure S4: Raman result of the graphene channels.
