# Supplementary material for: Performance Improvement of Residue-Free Graphene Field-Effect Transistor Using Au-Assisted Transfer Method
Source: Sensors (Basel). 2021 Oct 31;21(21):7262. doi: 10.3390/s21217262 (PMC8587746; doi:10.3390/s21217262)
Supplement: Supplementary file 1 [file sensors-21-07262-s001.zip › sensors-1393778-supplementary.pdf]

sensors MDPI

Supplementary Materials

Performance Improvement of Residue-free Graphene Field-Effect Transistor Using Au-assisted transfer Method

Yamujin Jang [1], Young-Min Seo [2], Hyeon-Sik Jang [2], Keun Heo [3] and Dongmok Whang [1,*]

[1] School of Advanced Materials Science and Engineering, Sungkyunkwan University, Suwon 16419, Korea; cyon24@skku.edu
[2] Institute of Advanced Composite Materials, Korea Institute of Science and Technology (KIST), Wanju 55324, Korea; ymseo88@kist.re.kr (Y.-M.S.); hsjang@kist.re.kr (H.-S.J.)
[3] School of Semiconductor and Chemical Engineering, Jeonbuk National University, Jeonju 54896, Korea; kheo@jbnu.ac.kr
* Correspondence: dwhang@skku.edu; Tel.: +82-31-2907399; Fax: +82-31-2907410

1. Calculation of Electronic Property

Sheet resistance (R_{sh}) is calculated with the following equation:

$$R_{sh} = \frac{(V_{x2} - V_{x1})\,\mathrm{w}}{I \cdot d}$$

where w is the width of the hall bar-channel and d is the distance between the voltage leads X_2 and X_1. I is the current flowing between X_2 and X_1. Carrier density (n_s) values can be obtained from the following equation:

$$n_s = \frac{C_{ox}(V_g - V_{dirac})}{q}$$

C_{ox} is gate oxide capacitance, and q is Coulomb unit charge.
Based on these values, mobility can be obtained using the following equation

$$\mu = -\frac{1}{n_s e R_{sh}}$$

In our measurements, the channel width (w) of a hall bar device was 5 µm, and the length between X_2 and X_1 was 20 µm. Devices were measured at room temperature with high vacuum conditions of 1×10^{-6} Torr. And the mobility distribution is at $n_s = 3 \times 10^{11}$ cm^{-2}.

Result section

1. XPS spectra of transferred graphenes

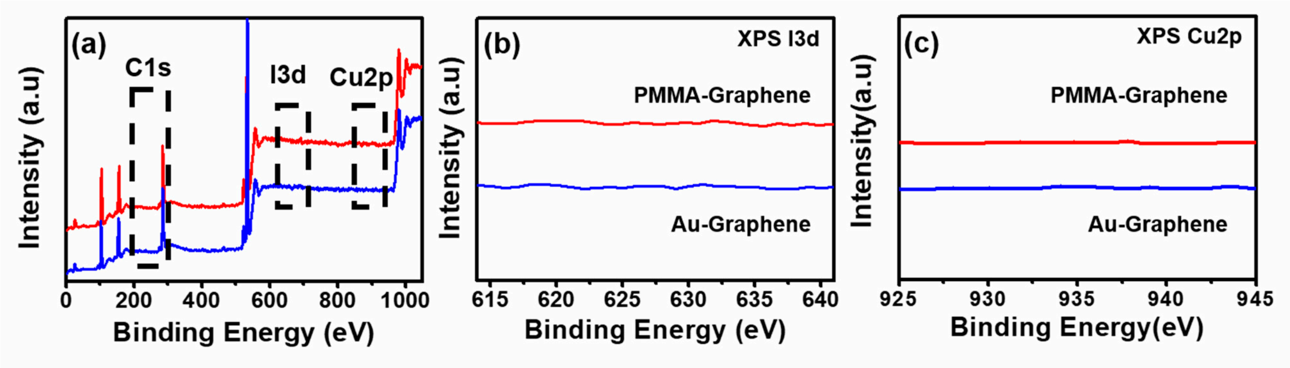

Figure S1. (a) XPS results for graphene surfaces transferred using the Au film-assisted and the PMMA-assisted methods. b) I_{3d} region in (a). Neither graphene transferred using PMMA or using Au film shows peaks related iodine. Graphene transferred using Au film shows similar results to those of PMMA-transferred graphene, which has no contact with iodine suggesting that Iodine molecules are removed completely without any effect on the graphene. (c) Cu_{2p} region in (a). Neither graphene transferred using both method shows peak related Cu. In Cu etching process, the Cu film completely etched without any Cu under the graphene.

2. Optical and SEM images

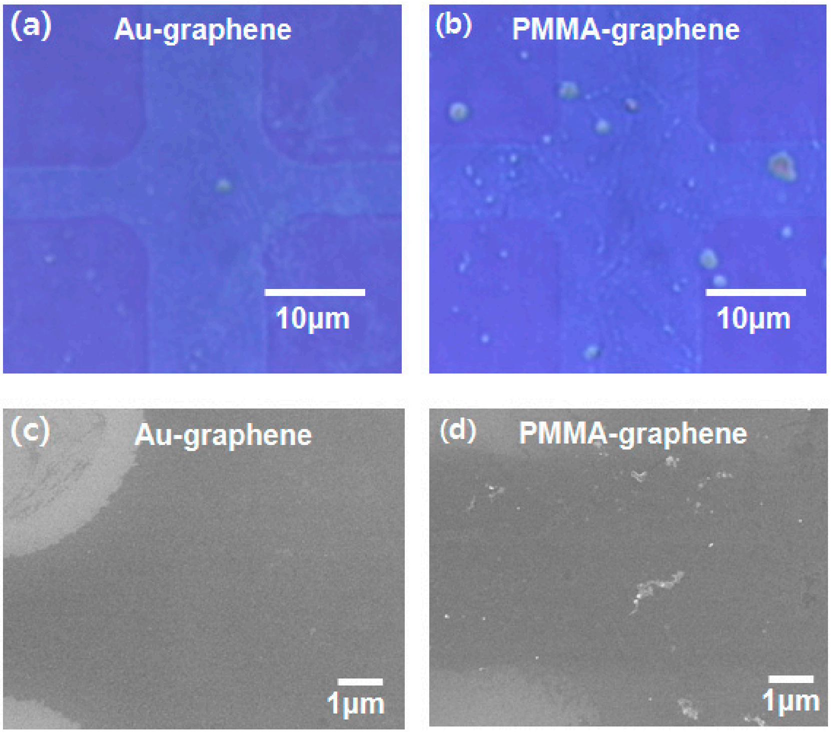

Figure S2. (a) and (b) Optical images of GFET channels fabricated using PMMA and Au supporting layers. The channel of graphene using Au has a clean surface; however, the channel of graphene using PMMA has residues on the surface. (c) and (d) SEM images of the same regions in optical images. In the SEM image, the surface of Au film assisted graphene shows a clean surface (Fig. S1(c)). However, PMMA assisted graphene has organic residues on the surface (Fig. S1(d)).

3. GFET Surface AFM Images

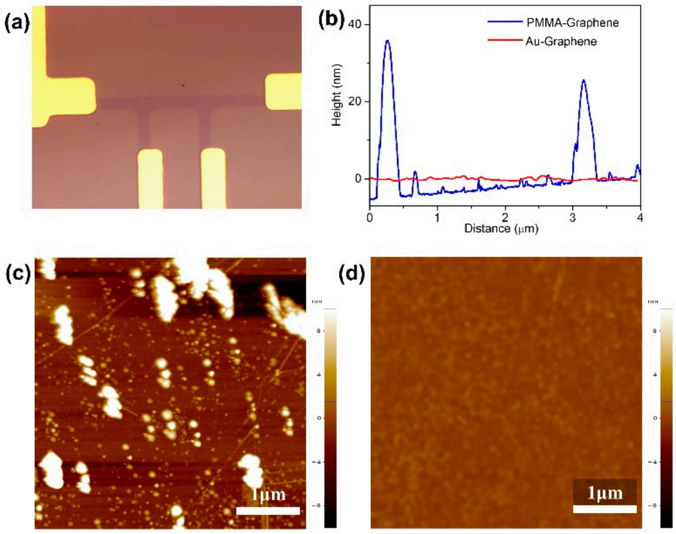

Figure S3. (a) Optical image of GFET channel area. (b) Line profile of graphene channel of GFETs fabricated using PMMA and Au film assisted transfer. AFM image of graphene channel area of GFETs fabricated using (c) PMMA and (d) Au-assisted transfer methods. The surface of PMMA assisted graphene is contaminated by organic residues. However, the channel area of Au-assisted graphene has no organic residue on the surface.

4. Raman Results

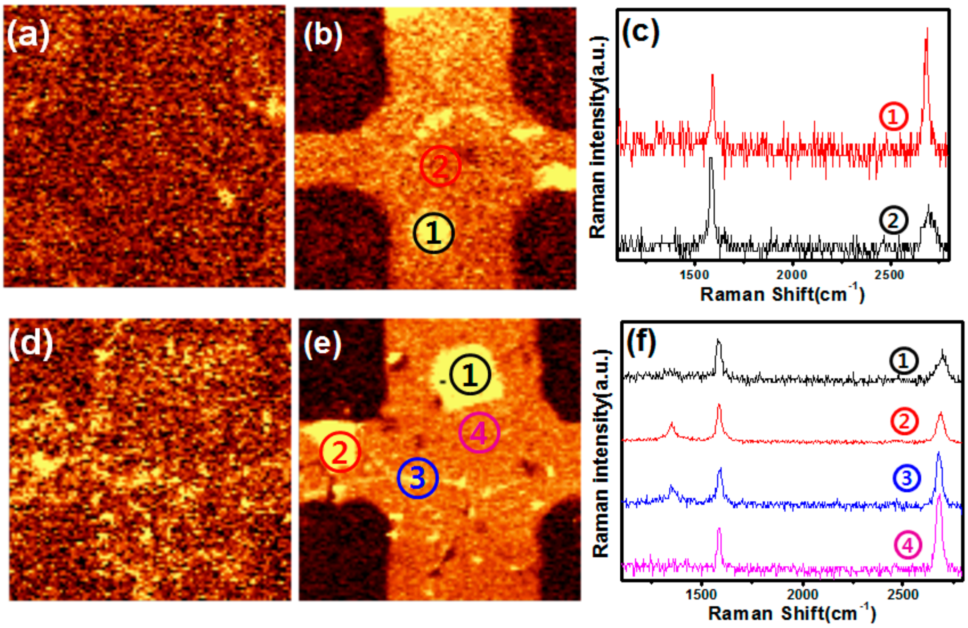

Figure S4. (a) and (d) Raman mapping images of D peak at the graphene channels fabricated using Au and PMMA, respectively. Graphene transferred using PMMA shows a high D peak in comparison with graphene transferred using Au. (b) and (e) G peak of both graphene channels. Bright regions in the images of G peak denote bilayers. (c) and (f) Raman spectra at marked regions of (b) and (d), respectively. Graphene using Au is divided into two parts, monolayer (② in (c)) and bilayers, and both of them have no defect (① in (c)). On the contrary, graphene using PMMA is divided into four parts, clean monolayer (④ in (f)), monolayer with many defects (③ in (f)), clean bilayer (① in (f)), and bilayer with defect s(② in (f)).
